# Dataset of differential lipid raft and global proteomes of SILAC-labeled cystic fibrosis cells upon TNF -α stimulation

**DOI:** 10.1016/j.dib.2016.08.012

**Published:** 2016-08-20

**Authors:** C. Chhuon, I. Pranke, F. Borot, D. Tondelier, J. Lipecka, J. Fritsch, M. Chanson, A. Edelman, M. Ollero, I.C. Guerrera

**Affiliations:** a Proteomic Platform Necker, PPN-3P5, Structure Fédérative de Recherche SFR Necker US24, 75015 Paris, France; b Institut Necker Enfants Malades, INSERM, U1151, Paris, France; c The CPN Proteomics Facility – 3P5, Center of Psychiatry and Neuroscience, UMR INSERM 894, 75014 Paris, France; d Geneva University Hospitals and University of Geneva, 1211 Geneva, Switzerland; e Institut Mondor de Recherche Biomédicale, INSERM, U955, and Université Paris Est Créteil, 94010 Créteil, France

## Abstract

Cystic fibrosis (CF) is a genetic disease due to mutations in the cystic fibrosis transmembrane regulator (CFTR), F508del-CFTR being the most frequent. Lipid raft-like microdomains (LRM) are regions of the plasma membrane that present a high cholesterol content and are insoluble to non-ionic detergents. LRM are essential functional and structural platforms that play an important role in the inflammatory response. CFTR is a known modulator of inflammation in LRM.

Here we provide mass spectrometry data on the global impact of CFTR mutation and TNF-a stimulation on the LRM proteome.

We used the Stable Isotope Labeling by Amino Acids in Cell Culture (SILAC) approach to quantify and identify 332 proteins in LRM upon TNF-a stimulation in CF cells and 1381 for the global proteome. We report two detailed tables containing lists of proteins obtained by mass spectrometry and the immunofluorescence validation results for one of these proteins, the G-protein coupled receptor 5A. These results are associated with the article “Changes in lipid raft proteome upon TNF-α stimulation of cystic fibrosis cells” (Chhuon et al., in press [1]).

**Specifications Table**

**Table:** 

Subject area	*Biology*
More specific subject area	*Proteomics of Cystic Fibrosis*
Type of data	*Figure, Tables*
How data was acquired	*Data dependent acquisition on a LTQ Orbitrap velos (Thermo Scientific) coupled to a nanoUltra High Performance Liquid Chromatography (UPLC) system (RSLC U3000, Thermo Scientific)*
	*Images were captured using Leica TCS SP5 AOBS confocal microscope (Heidelberg, Germany), equipped with 63x/1.4 oil differential interference contrast λ blue PL APO objective.*
Data format	*Filtered, analyzed*
Experimental factors	*Induced expression of wt-CFTR and F508del-CFTR in HeLa cells.*
Experimental features	*Populations of wt-CFTR and F508del-CFTR HeLa cells were labeled using SILAC and treated or not with 100 U/mL TNF-α. Extracts were mixed 1:1 and LRM were isolated with a sucrose gradient, digested in solution with trypsin and subjected to LC–MS/MS analysis.*
	*For total proteome, subcellular fractionation was performed, all fractions were acquired separately and analyzed together.*
Data source location	*Paris, France*
Data accessibility	*Data are available in this article.*

**Value of the data**

• We provide a list of LRM proteins purified on a sucrose gradient from CF cells.
• This experimental design allows multi-dimensional differential proteomic analysis: LRM protein recruitment as a function of inflammation stimulus and CFTR mutation.
• Confocal microscopy analysis provides differential localization of one representative LRM protein, depending on pro-inflammatory stimulus and CFTR mutation.

## 1 Data

We isolated LRM proteins by ultracentrifugation using a sucrose gradient. We validated the LRM proteins enrichment by Western blot analysis (Fig. 2a). We also performed subcellular fractionation on CF cells and all fractions were analyzed for total proteome.

The dataset contains two lists of proteins obtained with liquid chromatography coupled to high-resolution tandem mass spectrometry (LC–MS/MS) analysis of LRM (Supplementary Table II) with or without TNF-α stimulation and total proteome (Supplementary Table I) of cells.

## 2 Experimental design, materials and methods

**Fig. 1:**
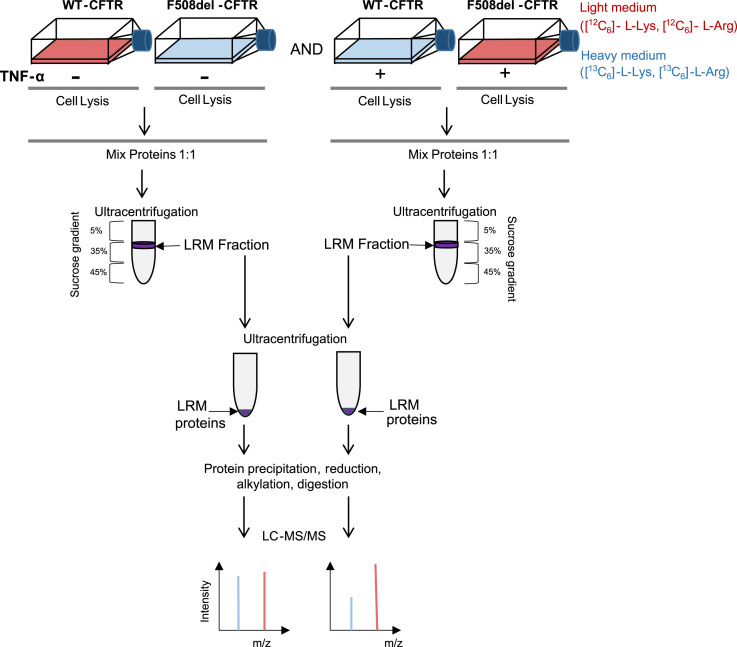
Experimental Workflow for LRM isolation and analysis. Populations of wt-CFTR and F508del-CFTR HeLa cells were labeled using SILAC and treated or not with 100 U/mL TNF-α. Extracts were mixed 1:1 and LRM were isolated with a sucrose gradient, digested in solution with trypsin and subjected to LC–MS/MS analysis.

The origin of reagents and chemicals, as well as the origin and culture conditions of cells, SILAC experiments, have been described in the associated research article [1]. For SILAC experiments, a representative workflow is shown in Fig. 1. Information about construction of HeLa cells expressing normal and F508del-mutated CFTR can be found in [2].

### 2.1 Detergent-resistant LRM isolation

**Fig. 2:**
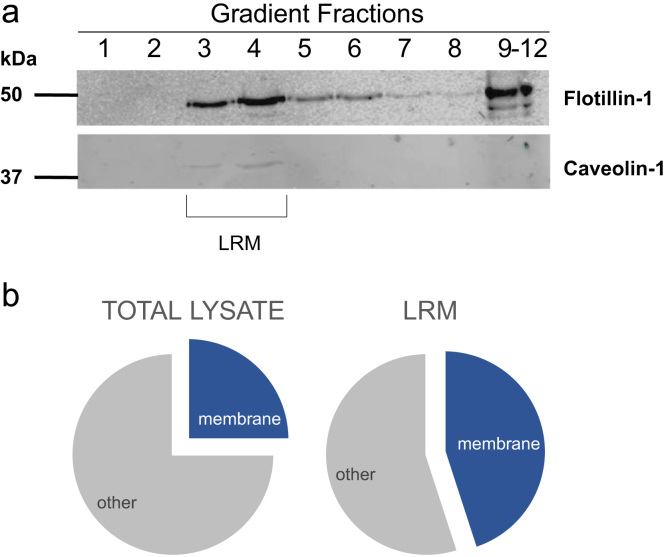
(a) Isolation of LRM fractions from HeLA cells. Twelve fractions (fractions 9–12 were pooled) were collected from the sucrose density gradient and analysed for marker proteins of LRM, caveolin-1 and flottilin-1, by western blot. LRM fractions are indicated. (b) Representation of proteins classified as membrane proteins in the total lysate and the LRM fraction, according to « GO cellular component slim » name « membrane ».

After SILAC experiments, cells were processed as described in [1]. After ultracentrifugation in a sucrose gradient, 12 fractions, including the light-scattering, fuzzy white band, were collected from the sucrose density gradient and 50 µL of each fraction was processed for western blot to test for the presence of LRM. The fractions enriched in LRM protein markers (mostly fractions 3 and 4, Fig. 2a) were diluted with 8 mL of ice-cold MBS and centrifuged for 3 h at 166,000*g* at 4 °C to pellet the detergent-resistant LRM.

### 2.2 Western blot analysis

Western blot analysis was performed on 50 µL of each fraction, except for fractions 9–12 that were pooled. Proteins were electrophoresed on a 10% SDS-PAGE and electrotransferred onto nitrocellulose membranes over 2 h in Tris–glycine buffer (Biorad, Hercules, CA) at 200 mA. Next, membranes were incubated in saturation solution: PBS+0.1% Tween 20 containing 1% non-fat dry milk (Regilait) and 1% bovine serum albumin (BSA) overnight at 4 °C. Proteins were immunoblotted for 2 h at room temperature with caveolin-1 antibody (dilution 1:500) and the flotillin-1 antibody (dilution 1:500). The secondary antibodies used were Odyssey IRDye goat anti-rabbit and mouse (Science-Tec, Courtaboeuf, France) diluted 1:5000 in 2% BSA with PBS+0.1% Tween 20 saturation solution. Caveolin-1 and flotillin-1 were detected using the Odyssey detection system (Li-Cor, Bad Homburg, Germany).

### 2.3 Subcellular fractionation

All procedures for fractionation were performed at 4 °C. Three T-75 flasks of wt-CFTR and F508del-CFTR HeLa cells were used for subcellular fractionation. All subcellular fractions were obtained by the method of Cox et al. [3]. Briefly, cells were washed two times with 6 mL ice-cold phosphate buffered saline (PBS) and once with 5 mL of ice-cold 250 mM sucrose, 50 mM Tris–HCl pH 7.4, 5 mM MgCl_2_, 1 mM DTT, 0.1 mM PMSF, 25 µg mL^−1^ spermine and 25 µg mL^−1^ spermidine (250-STMDPS buffer). Cells were homogenized (25–50 strokes) with a 1 mL Dounce. The lysate was centrifuged at 800*g* for 15 min and the supernatant was used for isolation of mitochondria, cytosol and microsomes, and the pellet for isolation of nuclei.

The nuclei pellet was resuspended in 500 µL of 250-STMDPS and re-homogenized for 1 min with a 1 mL Dounce and centrifuged at 800*g* for 15 min. The pellet was resuspended in 200 µL of 2 M sucrose, 50 mM Tris–HCl pH 7.4, 5 mM MgCl_2_, 1 mM DTT, 0.1 mM PMSF, 25 µg mL^−1^ spermine and 25 µg mL^−1^ spermidine and carefully placed on top of a 4 mL cushion of 2M-STMDPS, followed by ultracentrifugation at 80,000*g* for 35 min in a swinging-bucket rotor.

To extract nuclear proteins, pure nuclei were resuspended in 5 volumes of 20 mM HEPES pH 7.9, 1.5 mM MgCl_2_, 0.5 M NaCl, 0.2 mM EDTA, and 20% glycerol and incubated for 30 min with gentle rocking at 4 °C. Nuclei were lysed by 10 passages through an 18-gauge needle and centrifuged at 9000*g* for 30 min. The supernatant contained the nuclear soluble proteins and the pellet was resuspended in 5 volumes of 1% Triton, 1 mM DTT, 1 mM PMSF, 20 mM HEPES pH 7.9, 1.5 mM MgCl_2_, 0.5 M NaCl, 0.2 mM EDTA, and 20% glycerol and incubated for 30 min with gentle rocking at 4 °C, followed by lysis by 10 passages through an 18-gauge needle and centrifugation at 9000*g* for 30 min. The supernatant contained the nuclear membrane proteins.

Mitochondria were isolated by centrifuging the supernatant obtained after the first homogenization, at 6000*g* for 15 min. The mitochondrial pellet was resuspended in 10 volumes of 250-STMDPS and centrifuged at 6000*g* for 15 min. To extract the mitochondrial proteins, the pellet was resuspended in 0.5 mL of 10 mM HEPES pH 7.9, 1 mM DTT and 1 mM PMSF, incubated for 30 min on ice, sonicated to lyse mitochondria and centrifuged at 9000*g* for 30 min. The supernatant contained the mitochondrial soluble proteins and the pellet was resuspended in 0.5 mL of 20 mM Tris–HCl pH 7.8, 0.4 M NaCl, 15% glycerol, 1 mM DTT, 1 mM PMSF and 1.5% Triton X-100 (ME buffer) and centrifuged at 9000*g* for 30 min. The supernatant contained the mitochondrial membrane proteins.

Cytosolic proteins were isolated by centrifuging the supernatant obtained after the first centrifugation step of mitochondrial isolation, at 100,000*g* for 1 h in a swinging-bucket rotor. The supernatant contained the cytosolic proteins and the pellet contained the microsomal proteins. The microsomal pellet was resuspended in 0.5 mL of ME buffer, incubated for 1 h with gentle rocking and centrifuged at 9000*g* for 30 min. The supernatant contained the microsomal proteins.

### 2.4 Protein digestion, LC–MS/MS and data processing

All procedures are described in the associated research article [1].

### 2.5 Immunohistochemistry and confocal microscopy

All procedures are described in the associated research article [1].
