# Supplementary material for: Dataset of differential lipid raft and global proteomes of SILAC-labeled cystic fibrosis cells upon TNF -α stimulation
Source: Data Brief. 2016 Aug 20;9:51–6. doi: 10.1016/j.dib.2016.08.012 (PMC5011161; doi:10.1016/j.dib.2016.08.012)
Supplement: Supplementary file 1 — Supplementary material [file mmc1.pdf]

Pour de meilleurs résultats, ouvrez ce porte-documents PDF dans Adobe Reader X, ou dans Adobe Acrobat X, ou version ultérieure.

Télécharger tout de suite Adobe Reader
